# Orthodontic treatment as triggering factor of Medication Related Osteonecrosis of the Jaw in a breast cancer patient. Report of a rare case

**DOI:** 10.4317/jced.60046

**Published:** 2023-04-01

**Authors:** Evagelos Kalfarentzos, Agamemnon Chliaoutakis, Georgios Ntagiantis, Emmanouil Vardas, Panos Christopoulos

**Affiliations:** 1 Phd, Oral and Maxillofacial Surgeon, Department of Oral and Maxillofacial Surgery, Dental school, National and Kapodistrian University of Athens, Greece; 2 DDS, Postgraduate Student in Dentoalveolar Surgery, Department of Oral and Maxillofacial Surgery, Dental school, National and Kapodistrian University of Athens, Greece; 3 Assistant Professor in Hospital Dentistry, Department of Hospital Dentistry, Dental school, National and Kapodistrian University of Athens, Greece; 4 Assistant Professor in Oral and Maxillofacial Surgery, Department of Oral and Maxillofacial Surgery, Dental school, National and Kapodistrian University of Athens, Greece

## Abstract

**Background:**

Orthodontic treatment in adult patient is widely accepted nowadays. Therefore, orthodontists are needed to interact with more complex medical histories that may interfere with the orthodontic treatment. Antiresorptive medication is a widely used treatment for osteoporosis or malignancies that may affect the orthodontic movement and planning.

**Case presentation:**

A 53-year-old patient diagnosed with MRONJ one year after she started orthodontic treatment. Patients’ medical history includes breast cancer and treatment with high doses of denosumab for over 2 years. The patient had a drug holiday period in the start of orthodontic treatment and then resumed antiresorptive medication until extreme tooth mobility was observed during the orthodontic treatment. After a long absence from denosumab and failure of conservative means to control the established MRONJ we proceeded in surgical management of the affected area. After two relapse the patient is now stable and prosthetically rehabilitated.

**Discussion:**

The affected area was the only one treated orthodontically and in lack of other triggering factors such as extraction or acute inflammation we consider the orthodontic movement as triggering factor of MRONJ. BPs are widely known to affect orthodontic treatment as they suppress bone remodeling but there is a lack of literature as far as patients treated with denosumab or high doses of antiresorptive medication concern.

**Conclusions:**

Patients treated with high doses of antiresorptive medication should considered at high risk of developing MRONJ during orthodontic movement. Although, more studies are needed to establish a protocol for the patients seeking orthodontic treatment and treated with denosumab.

** Key words:**Medication Related Osteonecrosis of the Jaw, MRONJ, orthodontic treatment, bisphosphonates, denosumab, antiresorptive medication, surgical management.

## Introduction

Orthodontic treatment in adult patient has become widely accepted in most orthodontic practices. Adult patients have complicated medical or dental history more frequently than the average teenage patients undergone orthodontic treatment. Therefore, orthodontists must be aware of the risks, benefits, and effects of bisphosphonates and antiresorptive medication in use on the patient’s general health status, as well as on their orthodontic treatment outcomes. Most common reason of antiresorptive medication is osteoporosis but it is also administrated in high doses in several malignancies with bone metastasis involved. In accordance with the most recent literature, patients treated with intravenous high doses of bisphosphonates or denosumab are more likely to develop a serious complication in the jaws, described as Medication Related osteonecrosis of the Jaw (MRONJ) (1). The basic principles of orthodontic treatment are depending on the capability of the bone remodeling of each patient. Therefore, every condition or medication affecting bone remodeling may concern the orthodontist in order to modify or abort the execution of a treatment plan involving orthodontic movements (2).

## Case Report

A 53 years old female patient was presented in the Department of Oral Medicine & Pathology and Hospital Dentistry and referred to the Department of Oral and Maxillofacial Surgery of the Dental School of University of Athens, in January 2018, with chief complain of extreme tooth mobility in the mandible, periodical swelling in the gum area, difficulty chewing and pain in the anterior mandible. The patient also mentioned that the most of the symptoms started when shortly after the orthodontic treatment was initiated.

The patient was referred in our department due to the aggravated medical history and the need of surgical management of the hopeless teeth in the mandible.

Thorough medical and dental history was taken and revealed a number of conditions affecting the dental and oral surgery procedures. According to the patients’ medical file, she was diagnosed with breast cancer in July 2007. The patient undergone partial mastectomy and then she completed successfully six cycles of chemotherapy (3 cycles of 5 fluorouracil -epirubicin -cyclophosphamide and 3 cycles of docetaxel- epirubicin), radiotherapy and hormonal control until August of 2013. In September of 2015, bone metastasis in the spine were observed in a regular recall for the malignancy. The patient undergone radiotherapy in the lower spine and started chemotherapy with Vinorelbine, Cyclophosphamide, Exemestane until August 2018. The patient received antiresorptive agent (denosumab) from October 2015 until April 2017, medication with denosumab interrupted for three months and resumed until February 2018.

The patient also mentioned that she started orthodontic therapy in May 2017 in order to align the anterior teeth in the mandible, for better function and aesthetics. The orthodontic treatment was ongoing when the patient presented with extreme tooth mobility and swelling in the anterior mandible.

**Figure 1:**
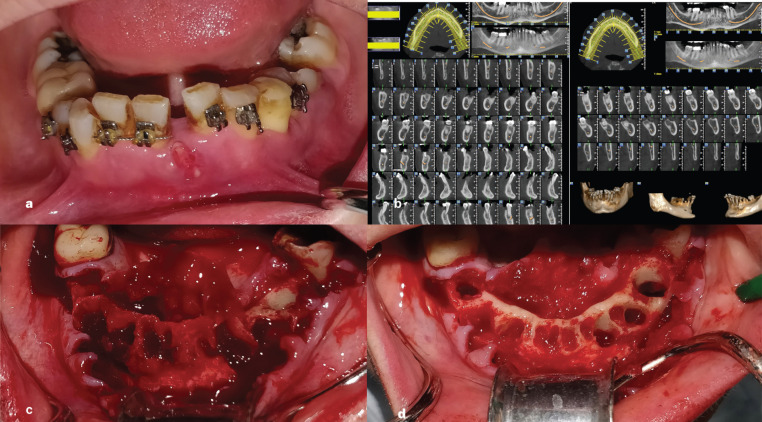
a: Initial clinical examination of the patient. Multiple fistulas in the anterior region observed. b: CBCT of the patient after clinical examination. Buccal perforations and extreme bone loss is noticed justifying the increased mobility of the teeth. c: Immediately after extractions and the raise of the flaps it is notable the buccal bone resorption and the excessive granuloma tissue in the area d: The surgical area after thorough debridement and smoothening of the sharp bony edges to enhance soft tissue healing.

Clinical examination of the patient showed multiple fistulae in the anterior region of the mandible, severe mobility of the teeth #35-45, pain in the chin area and progressive swelling of the anterior mandibular region (Fig. 1a). The swelling according to the patient was treated conservatively with antibiotic medication. After that the patient referred for radiological examination with Dental Scan. The CBCT of the patient revealed large radiolucent areas involving the root of the teeth #35-45 and several defects on the buccal cortical plate in the region, finding that are consisting with the clinical diagnosis of MRONJ (Fig. 1b).

The clinical and radiological findings in addition with patient’s medical history led us to the diagnosis of MRONJ stage II based on the staging system developed and updated by Ruggiero *et al*. in 2014. The patient was informed of her condition and the treatment plan proposed included extraction of the hopeless teeth and surgical management of the region in order to limit the expansion of the MRONJ. Absence of antiresorptive medication for the past 10 months was favorable for surgical management after 4 months (since September 2018) of irresponsive conservative treatment.

**Figure 2:**
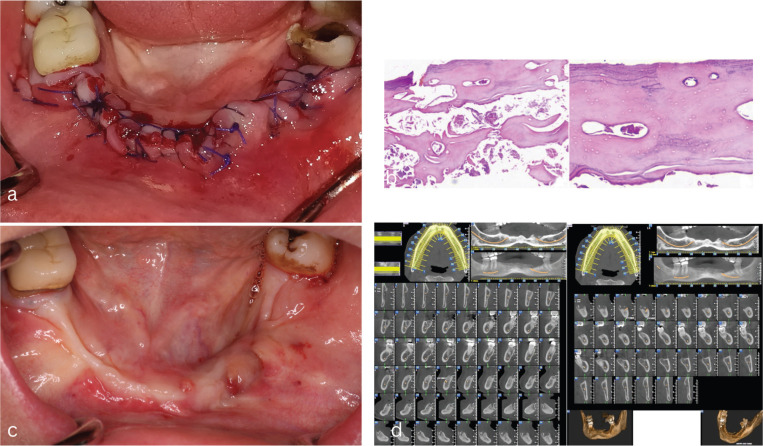
a: Suturing of the area and spare of tooth #36 due to lack of mobility. b: Histopathologic examination (Hematoxylin & Eosin original magnification x100): necrotic bone with bacterial colonies adherent in the bone surface-- empty osteocyte lacunae, reversal lines and absence of osteoblastic and osteoclastic activity. c: Clinical examination of the patient seven months after surgery revealed multiple fistulae in the left side. Patient also complained of mild pain in the region. d: Multiple sequestrum and a large radiolucent area lingually and in the middle of the alveolar ridge is revealed in the CBCT of the patient after seven months. The image is typical radiological image of MRONJ relapse.

The patient was informed of the complications of the surgery and also the high relapse rates and gave written consent in order to proceed to the first surgery in January of 2019. Under local anesthesia, a crestal incision was made and teeth #35-32 and #41-45 were extracted. A wide flap was developed in order to recognize the necrotic bone and remove the granuloma tissue surrounding the sockets (Fig. 1c). The bone removal stopped when vital, vascularized bone recognized clinically. All sharp bony edges were smoothened in order to facilitate optimum environment for soft tissue healing (Figure 1d). Releasing incisions were made in order to achieve primary closure. Two-layer suturing technique was used in order to achieve tension free closure of the flaps (Fig. 2a). Tooth number #36 was spared due to lack of mobility despite the moderate prognosis. A biopsy of soft and hard tissue was performed. Histopathologic examination showed necrotic bone with empty osteocyte lacunae, absence of osteoblastic and osteoclastic activity and bacterial colonies adherent to the necrotic bone surface (Fig. 2b). In addition, a dense and fibrotic connective tissue with diffuse inflammatory infiltration was also noticed. A final diagnosis of MRONJ was revealed.

Two weeks after surgery, failure to achieve primary closure of the soft tissue and exposed bone was observed in the area close to the tooth #36. Also increasing mobility and inability of restoration in this particular tooth was observed. Soft tissue was left for healing for another four weeks with no result. Therefore, a second localized surgical intervention was performed in order to extract tooth #36 and promote primary soft tissue healing in the region. The region was scraped until clinically vital bone was reached and granuloma tissue was removed from the sockets. The flaps sutured with double layer technique and covered the exposed alveolar ridge.

Two weeks later in the scheduled recall, soft tissue healing and integrity was excellent and no sign of inflammation observed. Regular recall of the patient every two months was scheduled and no prosthetic rehabilitation was advised.

Seven months later the patient complained about mild pain in the left mandibular area. Clinical examination revealed fistulas in the specific area (Fig. 2c). Antibiotics were subscribed in order to control the inflammation and radiological examination with CBCT was programmed. CBCT showed a large radiolucent area in the left side of the mandible matching the radiological findings of MRONJ (Fig. 2d). The patient was informed of the relapse and the need of a third surgical procedure.

**Figure 3:**
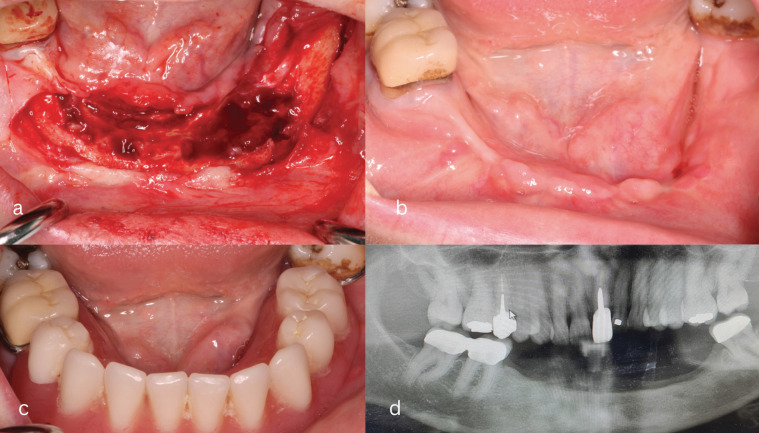
a: Clinical image after raising the flap during the last surgery. Extreme bone resorption in the left side is shown and massive granuloma tissue lingually and in the former sockets is observed. b: Soft tissue healing after six months of the last surgery. c: Prosthetic rehabilitation of the patient with specially designed removable denture in order to minimize the pressure on the mucosa. d: Radiological examination 3-years after first surgery showing no sign of defect or sequestrum.

Under local anesthesia, a large flap in the edentulous alveolar ridge was raised in order to debride thoroughly the region. Bone sequestrum and plenty of granuloma tissue were removed. The inflammatory granuloma tissue was discovered in all residual sockets and bony undercuts (Fig. 3a). It is noTable that minimal or not at all bone healing was observed even though the patient had the last dose of antiresorptive medication 14 months before the relapse. Deep periosteum releasing incision was made in order to cover the area with tension free flaps. Sutures were removed two weeks after the surgery and the patients had no relapse in the 13 months observation period followed (Fig. 3b). Nine months after the last surgery the patient was restored prosthetically with a removable denture (Fig. 3c). The restoration was designed in order tο minimize the pressure in the soft tissue. Trauma in the mucosa from dentures is a very common trigger factor of MRONJ that has to be eliminated in every case. Radiological examination 3-years after first surgery shows no sign of sequestrum or other defect in the mandible (Fig. 3d). Frequent recalls were programmed for the patient and thorough oral hygiene was performed periodically.

## Discussion

BPs and other antiresorptive drugs, such as denosumab, inhibit osteoclast differentiation and function and increase apoptosis, all leading to decreased bone resorption and remodeling (3). Osteoclast differentiation and function play a vital role in bone healing and remodeling in all skeletal sites, but ONJ occurs only primarily within the alveolar bone of the maxilla and mandible (1). On the contrary, successful orthodontic treatment depends on osteoclast activity. For a tooth to move, adequately functioning osteoclasts must be formed and present so that they can remove bone from the area adjacent to the compressed part of the PDL. Osteoblasts are also needed to form new bone on the tension side and remodel resorbed areas on the pressure side. The interruption of this cycle by antiresorptive medication such us BPs or denosumab, through osteoclast inhibition and reduced bone vascularization may affect orthodontic treatment by impeding tooth movement (4,5).

The role of BPs in orthodontic movement has been studied for multiple reasons, but denosumab has not been evaluated properly and the number of studies including the effect of denosumab in orthodontics are very few. In a study including patients medicated with BPs for osteoporosis, drug holiday for three months is suggested in order to reduce the risk of MRONJ. It is also mentioned that signs such us slower movement of the teeth is expected in long-use of BPs. Furthermore, if no drug holiday occurred, the clinician has to monitor the cases for signs such us extreme tooth mobility, sclerosis around teeth, obscured PDL, or excessive PDL space and alter or discontinue the treatment (6).

In a recent systematic review studying the effect of BPs in orthodontic treatment, it is mentioned that longer treatment times and lower movement rates are observed in the patients taking BPs. It is also mentioned that BPs can be used for anchoring a specific area with localized administration or stabilize an area after maxillary expansion or mandibular distraction in animal studies (7).

Denosumab, as a fully humanized antibody, blocks the receptor-mediated activation of osteoclasts and has no binding affinity for the bone matrix. Therefore, unlike BPs , the antiresorptive effect of denosumab should be mostly dissipated within six months of drug holiday (1). Although, in our case the patient had stopped the antiresorptive medication after excessive tooth mobility noticed the MRONJ was established. The affected area was the only one treated orthodontically and in lack of other triggering factors such as extraction or acute inflammation we consider the orthodontic movement as triggering factor of MRONJ in our case. It is also noticeable, that six months after surgery and soft tissue integrity a relapse of MRONJ occurred despite the lack of any antiresorptive medication.

According to the recent literature, patients administrated with high doses of antiresorptive medication are almost 100 times more likely to develop MRONJ (1). Recent guidelines does not exclude patients treated with BPs from orthodontic treatment especially those taking low-doses (8). Although, a recent study consider patient taking high doses of BPs at high risk of MRONJ and the patient physician should be included in the decision of beginning orthodontic treatment (9,10). Informing the patient for the risk of developing a side effect such us MRONJ is of great importance (11).

## Conclusions

According to the recent literature, in our best of our knowledge, there are not any references or protocol about the effect of denosumab in orthodontic treatment. Patients treated with any antiresorptive medication seeking orthodontic treatment should not be excluded, but treated with caution and according to their specific medical history. High doses of antiresorptive medication can compromise the bone remodeling and therefore make orthodontic treatment a high-risk procedure. MRONJ is a well-known complication of BPs and denosumab especially in oncological patients. The role of inflammation and dentoalveolar procedures needing bone remodeling has been established as a triggering factor of MRONJ. Therefore, orthodontics might trigger MRONJ when the patient’s bone turnover is altered by the use of antiresorptive medication. Although, the literature in the effect of denosumab in the various orthodontic treatment stages is very limited, more studies are needed in order to establish a certain treatment protocol.
